# Soil-Transmitted Helminth Eggs Are Present in Soil at Multiple Locations within Households in Rural Kenya

**DOI:** 10.1371/journal.pone.0157780

**Published:** 2016-06-24

**Authors:** Lauren Steinbaum, Sammy M. Njenga, Jimmy Kihara, Alexandria B. Boehm, Jennifer Davis, Clair Null, Amy J. Pickering

**Affiliations:** 1 Civil and Environmental Engineering, Stanford University, Stanford, California, United States of America; 2 Eastern and Southern Africa Center of International Parasite Control, Kenya Medical Research Institute (KEMRI), Nairobi, Kenya; 3 Mathematica Policy Research, Princeton, New Jersey, United States of America; 4 Innovations for Poverty Action, New Haven, Connecticut, United States of America; UMASS Medical School, UNITED STATES

## Abstract

Almost one-quarter of the world’s population is infected with soil-transmitted helminths (STH). We conducted a study to determine the prevalence and location of STH—*Ascaris*, *Trichuris*, and hookworm spp.—egg contamination in soil within rural household plots in Kenya. Field staff collected soil samples from July to September 2014 from the house entrance and the latrine entrance of households in Kakamega County; additional spatial sampling was conducted at a subset of households (N = 22 samples from 3 households). We analyzed soil samples using a modified version of the US Environmental Protection Agency (EPA) method for enumerating *Ascaris* in biosolids. We found 26.8% of households had one or more species of STH eggs present in the soil in at least one household location (n = 18 out of 67 households), and *Ascaris* was the most commonly detected STH (19.4%, n = 13 out of 67 households). Prevalence of STH eggs in soil was equally likely at the house entrance (19.4%, N = 67) as at the latrine entrance (11.3%, N = 62) (p = 0.41). We also detected STH eggs at bathing and food preparation areas in the three houses revisited for additional spatial sampling, indicating STH exposure can occur at multiple sites within a household plot, not just near the latrine. The highest concentration of eggs in one house occurred in the child’s play area. Our findings suggest interventions to limit child exposure to household soil could complement other STH control strategies.

## Introduction

Globally, about 1.5 billion people are infected with at least one species of soil-transmitted helminth (STH) [1]. STH are parasitic worms that live in the intestines of humans and other animals. The three predominant STH that can infect humans are *Ascaris lumbricoides*, *Trichuris trichiura*, and hookworm species (*Ancylostoma duodenale* and *Necator americanus*). *Ascaris* is the most prevalent, with an estimated global burden of 820 million infections [1]. Southeast Asia and Sub-Saharan Africa have the highest prevalence of all STH infections [1]. Both *Ascaris* and *Trichuris* are transmitted through the fecal-oral pathway, which includes transmission via water, hands, food, soil, and fomites. Hookworm species are transmitted transdermally, when infective larvae penetrate the skin [2]. However, *Ancylostoma duodenale* can also be transmitted if larvae are ingested [3]. Helminth infections can have severe health implications, such as malnutrition, anemia, vitamin deficiencies, stunted growth, and intestinal blockages [3]. Globally, the health impacts of STH infections cause an estimated 5.2 million disability-adjusted life years (DALYs) [1].

An emerging topic of research is control of STH contamination in the environment [4,5]. Soil is the primary environmental reservoir for STH eggs prior to transmission. In order to become infective, *Ascaris* eggs must incubate at 5 to 38°C for 8 to 37 days and *Trichuris* must incubate at 5 to 38°C for 20 to 100 days [2]. Hookworm eggs require 2 to 14 days of incubation at temperatures under 40°C to become infective larvae [2]. Past field studies have found soil contaminated with STH in both rural and urban settings with prevalences ranging from 11% to 82%, indicating significant geographic variability [4,6–12]. However, little work has been done to monitor environmental levels of helminth eggs in settings with ongoing mass drug administration programs to control STH. In addition, few studies have examined spatial variation of STH contamination within households and yards [4,7,13]. Identifying hotspots of STH egg contamination in the domestic environment would be valuable for the design and evaluation of interventions aimed to reduce transmission of STH.

This study characterized the concentrations of soil-transmitted helminths in soil within rural households and compared STH concentrations at the house entrance with those at the latrine entrance. We further assessed the spatial distribution of STH eggs within a small subset of households by analyzing soil samples from additional locations within the household plot. Using household survey data and soil samples, we also evaluated the associations between household, latrine, and soil characteristics and the presence of STH in soil. The study took place in rural Kenyan communities where an ongoing school-based national deworming campaign had been in effect for several years [14].

## Materials and Methods

We collected soil samples and household survey data from 67 households in rural villages of Kakamega County, Kenya (0.2833° N, 34.7500° E) from July to September 2014. The study area has an average temperature range from 17 to 30°C and an annual precipitation of 75 inches per year; these conditions are ideal for helminth transmission. A recent study found the prevalence of STH infection in school-age children in Western Province, which contains Kakamega County, was 25.1% hookworm, 24.2% *Ascaris*, 5.8% *Trichuris* [14]. We selected study households based on geographic location, as we wanted all households to be in close proximity to a field processing laboratory. All households had at least one child under the age of 3 years.

A local enumerator, who spoke Kiswahili and the predominant tribal language of the area (Kiluhya), collected soil samples and performed household surveys. The enumerator interviewed the mother of the household; if the mother was not present, she interviewed the father or other head of household. Survey data were collected electronically (Samsung Galaxy Tablet, Samsung Electronics, Suwon, South Korea) using Survey CTO (Dobility, Cambridge, MA). The enumerator recorded household, latrine, and soil characteristics. Household characteristics included the number of household members, animal ownership, self-reported helminth infection among children in the past 6 or 12 months, self-reported deworming of school-age children within the past 6 months, and observation of children wearing shoes at the time of interview. Field staff used direct observation to record latrine characteristics including the presence of a roof, three walls, door, ventilation pipe, plastic or concrete slab, flies, visible water, urine, or feces on the floor, and a drop hole cover. Field staff measured soil temperature, observed sun exposure, presence of visible wetness, presence of trash, and presence of feces at the sampling location. Observed sun exposure at the sampling location was characterized as full sun, partial sun, or full shade when the sample was collected.

The study procedures were approved by the Stanford Institutional Review Board (Protocol Number 23310) and the Kenya Medical Research Institute (KEMRI) Ethical Review Committee (SSC Number 2271). Field staff obtained written consent from all study participants on a prior visit, as well as oral consent on the day of soil collection.

We collected soil samples from both the house entrance and the latrine entrance at 62 households, and we collected soil from the house entrance from an additional 5 households that did not have a latrine or soil near the latrine entrance. We sampled at the entrance to the latrine because we hypothesized that the latrine may be a source of STH contamination, and because past studies have found STH contamination near and inside latrines [4,8]. The latrine entrance was defined as the location that was directly adjacent to the doorway to the latrine, which allowed us to collect samples from different types of latrines, including those with plastic or concrete slabs. We collected samples from the house entrance because we hypothesized that it could be a good indicator of STH contamination throughout the household environment. An in-depth study of fecal contamination in rural Tanzanian homes found significant *E*. *coli*, enterococci, and *Bacteroidales* contamination at this location [15]. In addition, the house entrance could be easily identified as a sampling location present in all households. When there was a hard or rocky slab present directly in front of the house entrance, we collected soil adjacent to the slab.

The enumerator collected soil samples in a 532 mL sterile WhirlPak bag (Nasco, Fort Atkinson, WI) from a 30 cm by 30 cm area, as measured by a styrofoam template. Before sampling, she measured the temperature of the soil at the sampling location with a temperature probe (Hanna Instruments, Checktemp 1C Thermistor Thermometer, Woonsocket, RI). To collect soil, she scraped surface soil, from no more than 0.5 cm deep, with a clean metal spade that was wiped with ethanol and a paper towel in between each sample collection. She transported samples back to the field lab at ambient temperature in a larger plastic bag.

We revisited three households for additional sampling to assess spatial variation in STH egg concentrations across a household plot. We chose these households based on the high concentration of helminth eggs detected in their soil during the first sampling visit and respondent availability for a revisit. The enumerator collected one sample from the following locations in the household plot: inside the latrine (if it had a dirt floor), the latrine entrance, halfway between the latrine entrance and the household entrance, the household entrance, the kitchen entrance, the washing area, the bathing area, and the child’s play area. She collected and transported soil samples following the same procedures described above.

### Soil Processing

This study analyzed soil for the presence and concentration of soil-transmitted helminths, including *Ascaris lumbricoides*, *Trichuris trichiura*, and hookworm species. We extracted helminth eggs from soil using a method modified from the US EPA method for detecting and enumerating *Ascaris* in biosolids [16]. Samples were homogenized by shaking them vigorously in the WhirlPak bag for 10 seconds. Rocks and leaves were removed from samples by sieving soil through metal window screens (0.2 x 0.2 cm opening) that were procured locally. We washed screens thoroughly with a brush and soap detergent between uses. If samples were too wet to sieve through the screen, we removed rocks and leaves manually with forceps. A 15 gram aliquot of homogenized soil was placed into a clean 50 mL centrifuge tube for subsequent enumeration of STH eggs; a 5 gram aliquot was used to determine the moisture content of the sample.

To process soil samples for enumeration of STH eggs, we first added surfactant, 0.1% Tween 80 (Sigma-Aldrich, St. Louis, MO), to each 15 g sample up to 35 mL and vortexed on a low speed for 10 seconds. We rinsed the sides and cap of the tube with surfactant, added surfactant to the 45 mL line, and left the samples to soak overnight. The following morning, we poured each sample into a 1000 mL beaker, added additional surfactant to the 500 mL line, and mixed with a magnetic stir bar on a stirring plate for 10 minutes. Then, we poured each sample through a size 50 mesh sieve (stainless steel mesh, 300 micron, H&C Sieving Systems, Columbia, MD), rinsed the sample through the sieve with surfactant, and rinsed the bottom of the sieve with 0.1% Tween 80 to capture any eggs stuck after exiting the sieve. We washed the sieve with a brush and detergent between uses. We left the samples to settle for 2 hours, after which they were vacuum aspirated to remove excess water and surfactant. We poured each remaining sample into two 50 mL centrifuge tubes and centrifuged at 1000 x g for 10 minutes (ELMI, Swing-Out CM-CMT centrifuge with 4 x 50 mL rotor, Riga, Latvia). We poured off the supernatant, added 5 mL of zinc sulfate solution (ZnSO_4_ heptahydrate, 1.2 specific gravity) as a flotation solution, vortexed for 5 seconds at a medium speed, and added additional zinc sulfate solution to the 40 mL line. We centrifuged at 1000 x g for 10 minutes and then poured the supernatant through a fine 400 mesh sieve (stainless steel mesh, 38 micron, H&C Sieving Systems, Columbia, MD). We washed the sieve with soap detergent in between uses. We rinsed the contents of the sieve into a final, clean 50 mL centrifuge tube using distilled water. We centrifuged the final tube at 1000 x g for 5 minutes to settle the helminth eggs. We removed the supernatant using a clean 25 mL serological pipette until only 1 mL of sample remained. The 1 mL of liquid was then transferred onto a Sedgwick Rafter slide (glass slide with brass frame, 1 mL cell volume, Wildco, Yulee, FL) using a clean, plastic 2 mL serological pipette. A parasitologist with 20 years of experience identifying helminth eggs in stool examined each slide under a microscope under 10x magnification. One of the authors (LS) also counted each slide so that there were two egg counts for each slide. We resolved large (>10% difference between counts) discrepancies between the two counts discussion and recounting; we replaced the original data with the recounted data. We calculated an average of the two counts and performed all data analysis using the average egg count. The entire protocol took approximately 8 hours, including a 2-hour settling step, to process 6 to 8 samples.

We collected field duplicates for 13% (N = 17) of samples by collecting soil from the area immediately adjacent to the sampling location. We processed technical replicates for 8% (N = 10) of samples by processing a second aliquot from the same soil sample. We ran laboratory blanks (N = 4) by processing a sample of distilled water through all protocol steps to ensure that there was no cross-contamination between samples.

To determine the soil moisture content, a 5 gram aliquot of each sample was placed onto a clean aluminum tray and dried for 18 to 24 hours at 110°C in a gravity convection oven. The next day, samples were removed and left to cool for 10 minutes before lab staff measured the dry weight of the soil, following the ASTM D2216 method [17]. We used the moisture content to normalize results by the total mass of dry soil.

We tested the recovery efficiency of this method using soil samples seeded with *Ascaris* eggs (Excelsior Sentinel, Trumansburg, NY) in a laboratory at Stanford University. We added approximately 1000 eggs to 15 g of organic loam soil (Stanford University, CA) and homogenized by sieving out large rocks and leaves using a size 8 sieve (stainless steel, 2380 micron) and vigorously shaking in a container. Three 15 g seeded replicates were processed following the steps detailed above. The recovery efficiency was calculated by taking the difference between the number of eggs added prior to processing and the number of eggs remaining at the end of processing and dividing by the number of eggs added prior to processing.

### Statistical Analysis

McNemar’s test was used to compare prevalence of STH eggs at the house entrance with the prevalence at the latrine entrance. A Wilcoxon sign-rank test was used to compare the difference in STH concentration in soil at the house entrance and the latrine entrance, and Spearman rank correlation was used to assess the correlation between the two sampling locations. P-values less than 0.05 are considered statistically significant. We used univariate logistic regression to explore the association between the presence of STH in soil and the following variables: number of household members, presence of improved sanitation, presence of a school-aged child, self-reported deworming drug consumption by a school-aged child, soil moisture content, soil temperature at the time of sample collection, and the presence of full sun exposure on the sampling location at the time of sampling. We report associations with a p-value less than 0.2. All analysis was performed with STATA version 13.

## Results

The households enrolled in the study had an average of 5 household members (Table 1). The mean age of the youngest child was 4.6 months and most (71.6%) households also had a school-aged child between the ages of 3 and 18. The majority (85.1%) of households reported ownership of animals in their compound. The most common animal in the compound was chickens, with 54 households owning at least one chicken. Most latrines had a roof (95.2%) and 3 walls around the latrine (90.5%). Few (6.4%) latrines had ventilation pipes, and most (95.2%) latrines had flies present in the latrine. Also, few latrines (7.9%) had visible stool present on the floor of the latrine.

**Table 1:** Characteristics of Study Households (N = 67).

	Mean (SD)	No. (%)
Number of household members	5.3 (2.3)	
Age of youngest child in months	4.6 (1.8)	
Presence of past STH infection in school-aged child in households with a school-aged child^+^		12 (25.0%)
Presence of animals in compound		57 (85.1%)
Improved latrine (JMP defined)		35 (52.2%)
Presence of 3 walls around latrine *		57 (90.5%)
Presence of latrine door *		42 (66.7%)
Presence of latrine roof *		60 (95.2%)
Presence of ventilation pipe in latrine *		4 (6.4%)
Presence of latrine slab *		33 (52.4%)
Flies observed in/around latrine *		60 (95.2%)
Visible stool observed on latrine floor *		5 (7.9%)
Urine observed on latrine floor *		34 (54.0%)
Distance from the household entrance to the latrine entrance (m) *	34.0 (18.5)	

^+^N = 48

*N = 63

The soil temperature at the time of sample collection ranged from 18.4° to 36.8°C, with a mean temperature of 24.3°C (SD = 3.8). The mean moisture content across all sampling locations was 17.4%. Approximately one-quarter of the sampling locations (26.1%) were exposed to full sunlight at the time of sampling. The soil characteristics were similar at both sampling locations (Table 2).

**Table 2:** Soil Characteristics of Sampling Location (N = 128).

	House Entrance		Latrine Entrance	
Variable	Mean (SD)	No. (%)	Mean (SD)	No. (%)
Soil temperature at sampling time (°C)	24.4 (4.2)		24.3 (3.3)	
Percent soil moisture content (%)	15.3 (16.0)		19.7 (27.0)	
Presence of trash		46 (68.7%)		45 (73.8%)
Presence of visible stool		0 (0%)		1 (1.6%)
Presence of full sun exposure		17 (25.4%)		18 (26.9%)

The egg recovery efficiency of our method was determined to be 37.2% (SD = 3.3%). The lower detection limit was 0.067 eggs/g soil (i.e. 1 egg in 15 grams of soil). We found no STH eggs in blank samples (N = 4). Duplicate field sample STH concentrations were not well correlated (r = -0.035), with a mean absolute difference of 11% (0.13 eggs/g dry soil, N = 17). Technical replicates were well correlated (r = 0.998), with a mean absolute difference of 18% (0.3 eggs/g dry soil, N = 9), excluding one outlier that had an absolute difference of 0.39 eggs/g dry soil (1 versus 6 eggs counted).

The overall prevalence of at least one STH egg in all samples was 15.5%. *Ascaris* was the most prevalent STH in all samples (11.6%), followed by *Trichuris* (4.7%) and hookworm spp. (0.8%) (Table 3). The prevalence of STH soil contamination in at least one location within a household was 26.8%, and *Ascaris* was the most commonly detected STH at households (19.4%). Prevalence of any STH egg in soil was slightly higher at the house entrance (19.4%, N = 67) than the latrine entrance (11.3%, N = 62, Table 3), but the difference was not statistically significant (McNemar’s test, p = 0.41). Among all positive samples, the mean STH concentration was 0.57 eggs/g dry soil and the median STH concentration was 0.23 eggs/g dry soil. The mean STH concentration of positive samples was 0.70 eggs per gram of dry soil (median = 0.24 eggs/g dry soil) at the house entrance and 0.33 eggs per gram of dry soil at the latrine entrance (median = 0.23 eggs/g dry soil); this difference was not statistically significant (Wilcoxon sign-rank, two-tailed, N = 62, p = 0.20). There was no correlation between soil contamination at the house entrance and at the latrine entrance (Spearman rank correlation = 0.15, p = 0.24) at households where both samples were collected; only two households (3.0%) had positive samples at both the household entrance and the latrine entrance.

**Table 3:** Prevalence and Concentration of STH in Household Soil in Kakamega, Kenya.

	Any STH			*Ascaris*			*Trichuris*			Hookworm spp.		
	n	%	Mean of positive samples	n	%	Mean of positive samples	n	%	Mean of positive samples	n	%	Mean of positive samples
**All samples (N = 129)**	20	15.5	0.57	15	11.6	0.71	6	4.7	0.11	1	0.8	0.07
**House entrance (N = 67)**	13	19.4	0.70	9	13.4	0.94	5	7.5	0.12	1	1.5	0.07
**Latrine entrance (N = 62)**	7	11.3	0.33	6	9.7	0.38	1	1.6	0.09	0	0.0	0.00

We found helminth eggs in multiple locations during spatial sampling at three household plots. At two of the three households, the locations with the highest concentration of eggs were the kitchen entrance and the bathing area. At one household, the highest concentration was found at the child’s play area (Table 4). We also found that two households had a similar range of concentrations [0, 0.49 eggs/dry g], whereas one household had a larger range of concentrations [0, 3.07 eggs/dry g] (Table 4).

**Table 4:** Concentration of Soil-Transmitted Helminth Eggs by Household Plot Location (N = 22 samples, from 3 separate households).

	Concentration of STH in Soil [eggs / g dry soil]								
	Inside the latrine structure	Latrine entrance	Halfway between latrine entrance and house entrance	House entrance	Kitchen area	Washing area	Bathing area	Child’s play area	Min-Max (SD)
**House 1**	0.30	0.31	0.08	0.08	0.49	0.16	0.40	0.00	0–0.49 (0.17)
**House 2**	n/a	0.00	0.00	0.00	0.15	0.00	0.08	0.38	0–0.38 (0.14)
**House 3**	n/a	0.00	0.16	0.31	1.28	0.08	3.07	0.15	0–3.07 (1.12)

We looked at the associations between household and soil characteristics and the presence of any STH eggs in soil using univariate regression. Self-reported infection of a school-aged child within the past 6 months was associated with increased odds of STH contamination (OR = 3.44, p = 0.052) and self-reported deworming of a school-age child may be associated with decreased odds of STH contamination (OR = 0.45, p = 0.198). The presence of full sun exposure over the sampling location at the time of sampling significantly decreased the odds of STH eggs in soil (OR = 0.12, p = 0.046). Number of household members, presence of trash at the sampling site, presence of improved sanitation, soil temperature, and soil moisture content were not associated with the presence of STH eggs in soil.

## Discussion

Our findings suggest STH soil contamination is present within households in rural Kenya, a setting with high coverage of on-site sanitation facilities (*e*.*g*. pit latrines) and an ongoing national school-based deworming campaign [14]. 26.8% of households had soil contaminated with at least one species of STH at either the house entrance, the latrine entrance, or both soil sampling locations. The prevalence and mean concentration of soil-transmitted helminths in soil near the latrine was lower than in a recent study in Tanzania (11.3% versus 71%; 0.33 eggs/g dry soil versus 1.5 eggs/g soil) [8]. However, choice of sampling location (entrance to latrine *versus* directly next to the pit), study area, and the use of different field laboratory methods could partially explain these differences.

Our spatial sampling results indicate there are multiple sites of possible helminth transmission and exposure within the home. The latrine was not the primary exposure location in the household; the prevalence and concentration of STH in soil were not significantly different at the house entrance (19.4%, 0.70 eggs/g dry soil) and the latrine entrance (11.3%, 0.33 eggs/g dry soil). Our results agree with a study that looked at STH in soil in Brazilian households that found that the median concentration inside the house was 0.8 eggs/g and 0.6 eggs/g near the defecation site [13]. Our spatial analysis provides evidence that soil contamination can be pervasive within a household; the kitchen and bathing areas can contain more STH eggs than near the latrine. A study that looked at bacterial contamination of household soil in Tanzania found a similar trend; there was higher contamination inside the home and at the food preparation area than at the latrine [15]. One hypothesis is that STH eggs could enter the household on produce. A study in Kisii, Kenya, found that 65.5% of produce samples were contaminated with STH and other parasite eggs [18]. Washing produce reduces contamination, but the resulting rinse water is contaminated with parasite eggs and is often discarded on the ground [19].

One limitation of this study is the recovery efficiency of our method. There is no standard method for extraction of STHs from soil [20]; we based our method on the US EPA method for detecting and enumerating *Ascaris* in biosolids because of its potential for a high recovery efficiency. We reduced the overall processing time of the method by omitting two soaking and sedimentation steps, thus improving feasibility within a field lab setting. The resulting recovery efficiency of the method was 37.2% for *Ascaris*. This recovery efficiency is within the range of typical recovery efficiencies in published studies; the median recovery efficiency reported in a recent systematic review of methods to detect STH in soil was 25% [20]. A second potential limitation of the method is its ability to isolate hookworm larvae from soil. Hookworm larvae may not survive the length of the processing protocol. In addition, the infective, filariform larvae is approximately 600 μm long, and the first sieve used in the laboratory protocol has an opening of only 300 μm. It should be noted that our sampling methodology did not produce consistent field duplicates. However, the differences in field duplicates can be explained by spatial variability in STH egg concentration; sometimes it was not possible to collect duplicate samples from directly adjacent locations. Most households had chickens, which can be infected with *Ascaridia galli* and *Heterakis gallinarium*, which have eggs morphologically similar to *Ascaris*. However, our lab technicians were specifically trained to differentiate between these species’ eggs and *Ascaris* eggs using distinctive features (*e*.*g*. shape of the egg).

Detection of soil-transmitted helminth eggs in soil at almost one-third of households suggests that child exposure to household soil poses a substantial health risk. Notably, geophagy (soil eating), is common among school children in Kenya and other countries [21–25]. A study in western Kenya found that 73% of primary schoolchildren in the study practiced geophagy; most reported eating soil at least once a day. The estimated median soil consumed per day was 28 g, with a range of 8 to 108 g [21]. Geophagy has been shown to be associated with *Ascaris* and *Trichuris* infection for children in western Kenya, Zambia, South Africa, and Jamaica [22–24,26]. Considering the mean concentration of positive samples in our study was 0.57 eggs/g dry soil, children who practice geophagy are likely to ingest STH eggs regularly. When eggs are frequently ingested, deworming programs are unlikely to provide sustained reductions in STH infections. Additionally, in our spatial analysis, we found two of three households had STH eggs at the child’s play area, and the highest concentration of eggs in one household occurred at the child’s play area. Our findings suggest interventions to limit child exposure to household soil (*e*.*g*. promoting soil-free play areas, discouraging geophagy) could complement other STH control strategies, such as mass drug administration.

## Supporting Information

S1 Dataset Laboratory and survey data from Kakamega, Kenya. Prevalence, concentration, and risk factors for STH contamination in soil.
(ZIP)
